# Influence of Sodium Borohydride Content on Triangular Silver Nanoprisms Dropped on Copper Hydroxide Nanowire-Based SERS Substrates

**DOI:** 10.1021/acsomega.4c06818

**Published:** 2024-11-15

**Authors:** Daniela González-Zárate, José Luis Zamora-Navarro, María Beatriz de la Mora, Guillermo Santana-Rodríguez, Mario Díaz-Solís, Luis Zamora-Peredo

**Affiliations:** † Centro de Investigación en Micro y Nanotecnología, Universidad Veracruzana, Boca del Río, Veracruz 94294, Mexico; ‡ Instituto de Ciencias Aplicadas y Tecnología, Universidad Nacional Autónoma de México Ciudad Universitaria, Delegación Coyoacán, CDMX 04510, Mexico; § Instituto de Investigación en Materiales, Universidad Nacional Autónoma de México, Ciudad Universitaria, Delegación Coyoacán, CDMX 04510, Mexico; ∥ Facultad de Ciencias Químicas, Universidad Veracruzana, Boca Del Río, Veracruz 94294, Mexico

## Abstract

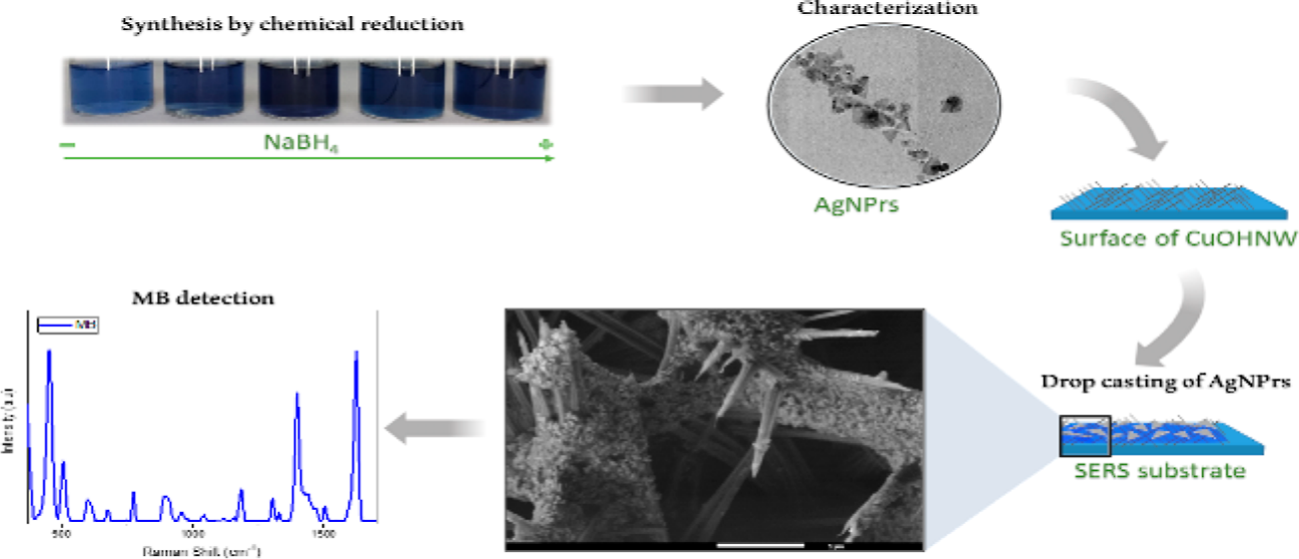

In this work, surface-enhanced Raman scattering substrates with triangular silver nanoprisms (AgNPrs) dropped on copper hydroxide nanowires (CuOHNWs) were evaluated. AgNPrs were synthesized in colloidal solution using Ag nitrate, polyvinylpyrrolidone, trisodium citrate dihydrate, hydrogen peroxide, and sodium borohydride (NaBH_4_). A set of five solutions with volume percentages from 0.99 to 4.76% v/v of NaBH_4_ as reducing agents was studied. The reaction time associated with blue coloration was determined. The evolution of the colloids was studied by UV–vis spectroscopy over a period of 42 days, confirming the good stability of AgNPrs. In addition, their colloidal stability was also verified by the zeta potential. The influence of NaBH_4_ concentration over the AgNPrs morphology was studied by scanning electron microscopy (SEM), transmission electron microscopy (TEM), and dynamic light scattering (DLS). Finally, SERS substrates were fabricated with AgNPrs deposited on CuOHNW and used to detect methylene blue as a probe molecule. AgNPrs obtained with 2.91% v/v NaBH_4_ presented the smallest sizes, and their SERS substrate presented the best Raman intensity.

## Introduction

Metallic nanomaterials have garnered significant attention both fundamentally and technologically due to their unique physical and chemical properties.^1^ These nanostructures offer a large surface area, which grants them superior optical, electronic, magnetic, and thermal properties compared to bulk materials, making them particularly interesting for sensing applications.^2^ Anisotropic nanoparticles, such as triangles, are intriguing due to their reduced symmetry, leading to unusual chemical and physical behaviors. Triangular gold (Au) and silver nanoprisms (AgNPrs) stand out for their structure- and medium-dependent optical properties. NPr have many advantages over other nanoparticles due to plasmonic response in a wide region of the electromagnetic spectrum, which includes the visible and near-infrared range. Because of their anisotropic morphology, the edges and vertices of the NPrs generate a stronger local concentration of the electric field,^3^ resulting in better sensitivity for sensors based on plasmonic resonance and surface-enhanced Raman scattering (SERS).^4^ Choosing Ag over Au depends on the application and required properties. However, in this work, AgNPrs were selected over Au nanostructures because, although Au offers good chemical stability and biocompatibility, Ag is superior in applications in which optical efficiency and higher sensitivity are needed. They can be produced in large quantities, and their dimensions can be controlled through physical, chemical, and biological methods. Triangular AgNPrs or nanoplates, which are distinguished by their triangular and flat shape, with small thickness, have been wide researched by their tunable surface plasmon resonance in the visible and near-infrared spectrum.^5^ Due to the unique characteristics, various studies have been conducted for different applications, including the design of a dual fluorimetric and colorimetric sensor,^6^ precise detection based on morphology transformation,^7^ detection of pesticides in seeds and crops to prevent diseases,^8^ rapid molecular diagnostics for SARS-CoV-2,^9^ detection of commonly used fluoroquinolone antibiotics in animal husbandry,^10^ multifunctional probes acting as signal captors and optical transducers,^11^ and evaluation of the anticancer properties of ovarian cancer in vitro and in vivo.^12^

Additionally, their use as highly sensitive SERS substrates for the detection of the chemotherapeutic compound *N*-acetyl procainamide (NAPA) through a photoinduced synthesis method,^13^ to mention a few. Colorimetric immunoassays are widely used for the detection of tumor markers using enzyme-linked ligands due to their simplicity and effectiveness. Dual-mode assays, which combine SERS and colorimetry, have been implemented to create a single detection system. This system ensures effective and sensitive detection of multiple samples, providing multiple output signals simultaneously, thereby enhancing the credibility of the results.^14−16^

Is it possible to obtain different morphologies of NPrs through one-pot synthesis, utilizing hydrogen peroxide (H_2_O_2_) as an oxidizing agent during the reduction of silver nitrate (AgNO_3_) by sodium borohydride (NaBH_4_), in the presence of trisodium citrate and polyvinylpyrrolidone (PVP) at room temperature?^17,18^ Using the same chemical reduction approach but with the addition of disodium succinate hexahydrate (DSSH), tiny and well-controlled NPrs have been obtained, which were subsequently coated with SiO_2_ to enhance their functionality, biocompatibility, and cell viability,^19^ large nanoplates have also been fabricated on a large scale using the same one-step synthesis method without citrate, although in this case it was necessary to add an antifoaming agent (A204).^20^ Numerous studies have been carried out evaluating the influence of citrate, PVP, AgNO_3_, or hydrogen peroxide^19−22^ content on the synthesis process and shape of AgNPr; however, few have studied the effects of NaBH_4_ content on their temporal stability.

In this work, the influence of NaBH_4_ concentration over the AgNPrs morphology was studied by scanning electron microscopy (SEM), transmission electron microscopy (TEM), and dynamic light scattering (DLS). The chemical stability of colloidal solution was monitored over 42 days by UV–vis spectroscopy. Besides, SERS substrates were prepared by a simple drop-casting method, where the AgNPr was deposited on the copper hydroxide nanowire (CuOHNW) surface. This technique allows us to get a uniform and controlled distribution of the NPrs over the nanowires. The effectiveness of this method is evaluated through morphological and chemical characterization of the deposited AgNPr. The results obtained show how the change in the volume of NaBH_4_ not only affects the morphology and size of the AgNPr but also their capability to enhance SERS sensitivity.

## Materials and Methods

The reagents used are as follows: sodium borohydride (NaBH_4_) (Meyer, ≥98%), hydrogen peroxide (H_2_O_2_) (Golden Bell, 30–35%), polyvinylpyrrolidone (PVP) (Sigma-Aldrich, wt 40,000), silver nitrate (AgNO_3_) (Meyer, ≥98%), trisodium citrate dihydrate (Na_3_C_6_H_5_O_7_·2H_2_O) (J.T. Baker, ≥99%), methylene blue (MB) (C_16_H_18_ClN_3_S·3H_2_O) (Hycel, ≥99%), and deionized water.

### Synthesis of Silver Nanoprisms

The synthesis of AgNPr was carried out following the method of Huang T. and Nancy Xu.^23^ Solutions with 30 mM of sodium citrate, 2% w/w of PVP, 0.11 mM of AgNO_3_, 200 mM of NaBH_4_, and 120 μL of H_2_O_2_ were prepared. In a beaker, 3.68 mL of sodium citrate, 3.68 mL of PVP, 0.12 mL of hydrogen peroxide, and 43 mL of AgNO_3_ were added, resulting in a 50.48 mL solution. Then, 0.99, 1.96, 2.91, 3.85, and 4.76% v/v of NaBH_4_ were added. A reaction volume of 30 mL was used. The solution was vigorously stirred at 600 rpm. During stirring, coloration changes were observed ranging from yellow to blue, indicating the formation and growth of AgNPrs.

### Characterization of AgNPr

For the absorption study, a Thermo Scientific Genesys 50 spectrophotometer was used, capable of exploring the UV and visible regions (190 to 1100 nm). For each measurement, a quartz cell was used with water as the blank. Measurements were taken at 1.0 nm intervals at a slow speed. Characterization by DLS and zeta potential (ZP) was performed using a Zetasizer Nano ZS90 size analyzer from Malvern Panalytical. This device uses a He–Ne laser at 633 nm with a power of 4 mW and an optical dispersion angle of 90°. The TEM study was carried out with a JEOL model JEM2010 FEG equipment in a carbon-coated copper cell with thin grids of the same material. SERS measurements were conducted using an Ocean Insights QE Pro Raman spectrometer with an excitation laser at 785 nm, a grating of 1200 lines/mm offering a spectral resolution of 14 cm^–1^, and an integration time of 3 s. A laser power of 139 mW was used. The spectral range covered by this equipment was from 150 cm^–1^ to 3000 cm^–1^. SEM was carried out using a JEOL JSM-7600F instrument, which has an acceleration voltage of 3.0 kV and operates at a working distance of 0.8 mm.

### SERS Substrate Preparation

The SERS substrate was prepared using a copper foil with CuOHNWs on the surface obtained by the anodization process; details can be found in an earlier report.^24^ Over the CuOHNW, 20 μL of AgNPr solution was dropped and dried for 24 h. Finally, 20 μL of an aqueous solution with MB were dropped and incubated 1 h before Raman measurements.

## Results and Discussion

**Figure 1:**
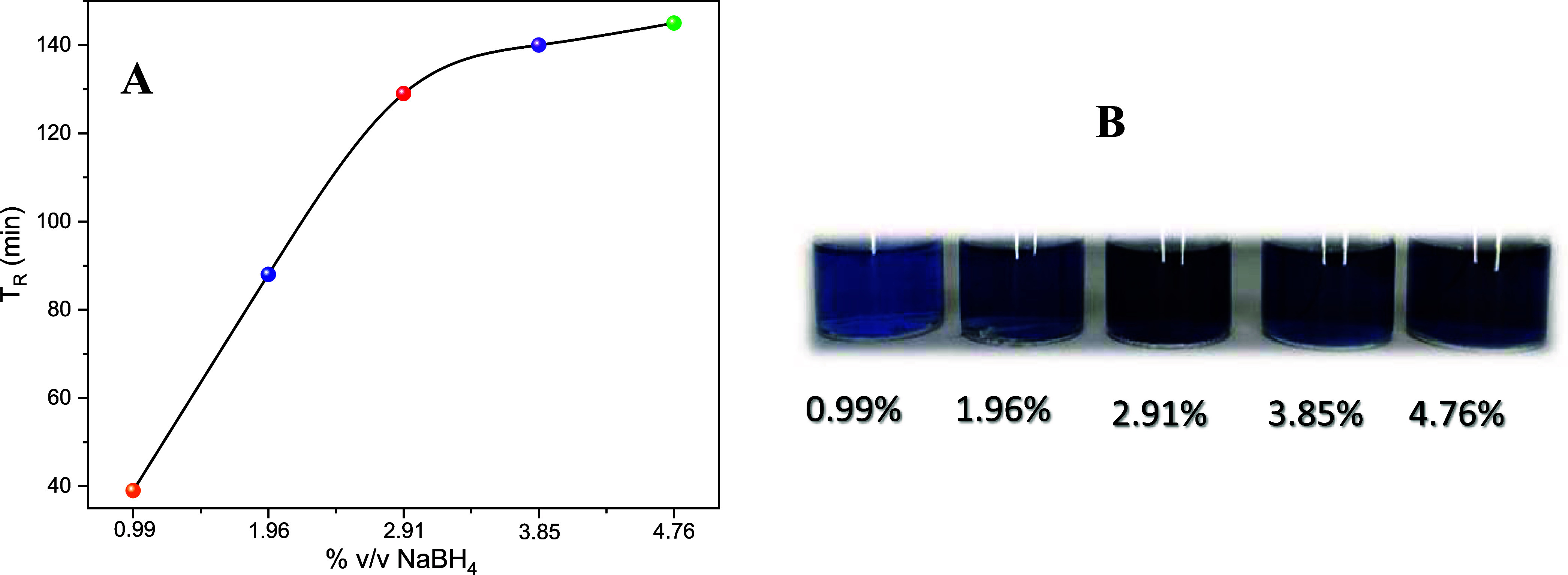
Behavior of the reaction time (A) and coloration (B) of the solutions with percentages of 0.99, 1.98, 2.91, 3.85, and 4.76% of NaBH_4_.

The formation of AgNPr was achieved by adding a % v/v of NaBH_4_ to the container with 30 mL of the stock solution (PVP, H_2_O_2_, AgNO_3_, and CT), which initially is colorless. PVP acts as a reaction controller, preventing agglomeration, while sodium citrate serves as a stabilizer, controlling the size of the nanostructures and aiding the anisotropic growth adsorbing more strongly to the Ag(111) surface to direct the final triangular shape. H_2_O_2_ acts as an oxidizing agent and plays a key role in the formation of the prisms, regulating their size and shape and promoting the formation of AgNPrs instead of nanospheres. After a vigorous stirring time, a spontaneous coloration process occurs (10–15 s), starting colorless, turning yellow, and finally turning blue.^25^ At that point, the chemical reaction time is considered complete. There is no nanostructure formation until the color change happens; in this case, the blue color indicates a triangular morphology. Therefore, the reaction time is measured from the moment that NaBH_4_ is added until the color change is observed. Figure 1A shows the behavior of the reduction time (measured from NaBH_4_ addition until the blue coloration) as the NaBH_4_ content increased from 0.99 to 4.76%. A linear relationship is observed until the NaBH_4_ content is 2.91%. For higher NaBH_4_ concentrations, saturation is occurring in the reaction due to an excess of reductant. This means that, at the beginning, with a smaller volume of NaBH_4_, there is still a considerable amount of precursor available for reduction. However, as the amount of reductant increases, the precursor begins to deplete, and even though more reducing agents are available, there is no longer enough precursor. At this point, the reaction time tends to be constant. Figure 1B shows the color of solutions after the reaction time where different coloration is observed due to the density and size of the AgNPrs; for lower density and greater size, the coloration is lighter; on the other hand, if the density is greater consequently, the size of the structures is smaller than the coloration is darker. This can also be corroborated by the absorption study.

**Figure 2:**
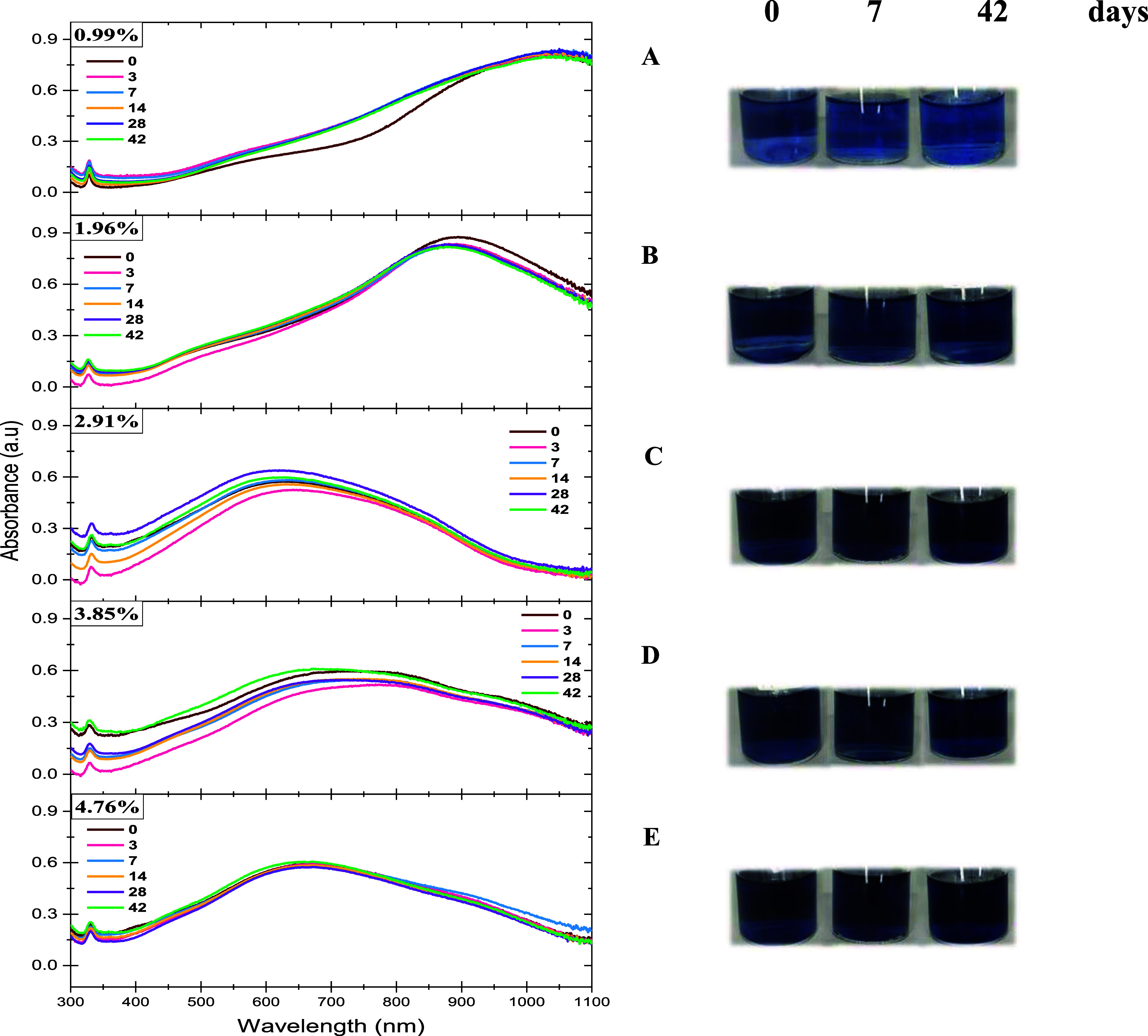
Absorbance spectra of colloid solutions synthesized with 0.99, 1.96, 2.91, 3.85, and 4.76% v/v of NaBH_4_ after 0, 3, 7, 14, 28, and 42 days. Photographs (A–E) correspond to each percent.

The absorbance spectra of solutions after the blue color was observed reveal localized surface plasmon resonances (LSPR) corresponding to the size, shape, and morphology of the nanostructures, indicating the presence of AgNPr, Figure 2. All spectra exhibit one sharp peak near 330 nm and more intense wide bands between 400 and 1100 nm, typical spectra associated with triangular structures of AgNPrs.^22^ In samples with 0.99 and 1.96%, there is a dominant wide band near 1000 and 885 nm, respectively, suggesting the biggest AgNPrs. When the NaBH_4_ content was 2.91, 3.85, and 4.76%, dominant absorption bands centered between 500 and 800 nm were observed, suggesting AgNPrs with middle and more dispersed sizes. Furthermore, the temporal stability of colloid solutions was evaluated by absorbance measurements for 42 days, and good stability was found. Photographs in Figure 2 show that the color of colloid solutions did not have considerable changes.

**Figure 3:**
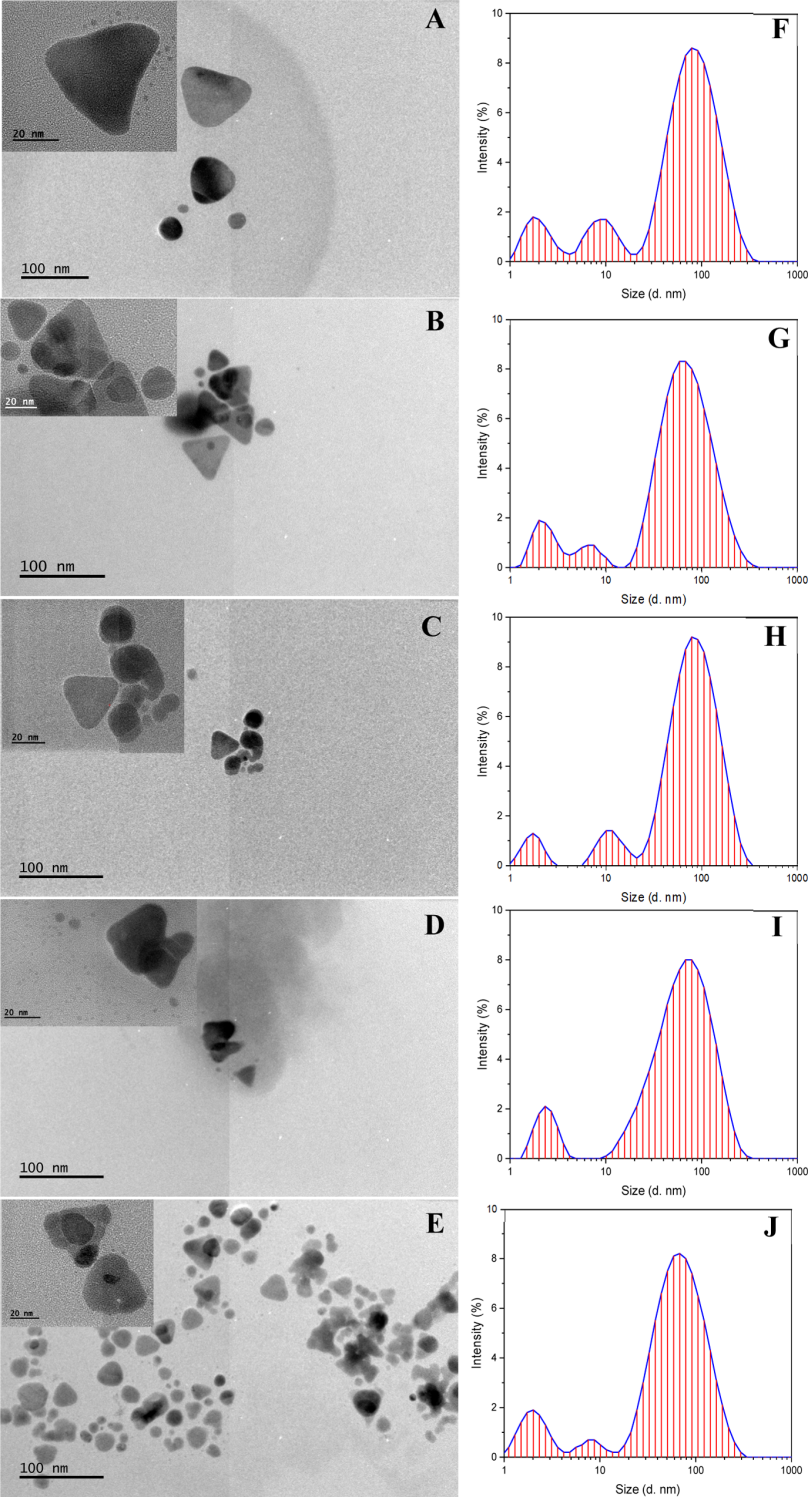
TEM micrographs (A–E) and DLS histograms (F–J) of AgNPr synthesized with 0.99, 1.96, 2.91, 3.85, and 4.76% v/v NaBH_4_, respectively.

TEM and DLS studies were used to evaluate the size distribution of AgNPr, as shown in Figure 3; NPrs obtained with 2.91% NaBH_4_ have the lowest and concentrated size values. Considering that the chemical reduction method used is polydisperse and initially the reduction of AgNO_3_ with NaBH_4_ form nanospheres. However, with the addition of H_2_O_2_, NPrs can be formed, indicating that during the nucleation and growth process, nanostructures of different morphologies, including nanospheres and NPrs, can be produced. The solutions have a high polydispersity index (PDI) originated by the intermediate morphologies of the AgNPr in agree with the broad absorption bands observed by the UV–vis study. ZP analysis was performed on neutral colloids at pH 7, revealing that the colloid with 2.91% NaBH_4_ had superior chemical stability, with AgNPr ranging in size from 60 to 80 nm, see Table 1. With TEM images of AgNPr synthesized with 0.99, 1.96, 2.91, 3.85, and 4.76% v/v of NaBH_4_ is possible to corroborate the different sizes and shapes correlated with the UV–vis and DLS spectra. It was possible to conclude that the colloids with lower % v/v NaBH_4_ were the AgNPr with larger sizes, which could be verified with the micrographs. On the other hand, the colloids with higher percentages of NaBH_4_ presented similar absorption bands, and by TEM it has been possible to identify that there are changes in morphology and size, this is because the reduction method is polydisperse and in the process of nucleation and growth can form nanostructures of different morphologies, including nanospheres and NPrs, as this justifies the change of coloration in the solutions.

**Table 1:** Diameters, Polydispersity Index (PDI), and Zeta Potential (ZP) of the AgNPr

% v/v NaBH_4_	hydrodynamic diameter (nm)	PDI	ZP (mV)
0.99	78.82 ± 10.06	0.627	–17.5 ± 0.5
1.96	78.82 ± 10.06	0.692	–18.8 ± 1.1
2.91	58.7 ± 9.36	0.69	–49.5 ± 0.8
3.85	68.06 ± 9.29	0.638	–20.7 ± 1.9
4.76	68.06 ± 9.29	0.671	–19.9 ± 3.5

**Figure 4:**
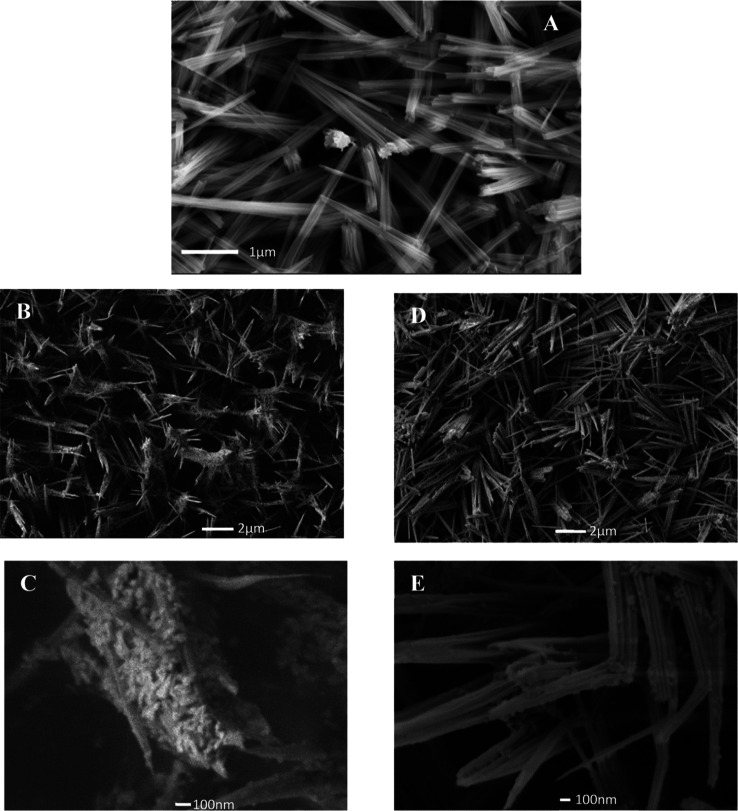
SEM images before (A) and after the AgNPr were deposited (B–E) over CuOHNW. NPr with 1.96 and 3.85% v/v of NaBH_4_ are showed in (B,C) and (D,E), respectively.

**Figure 5:**
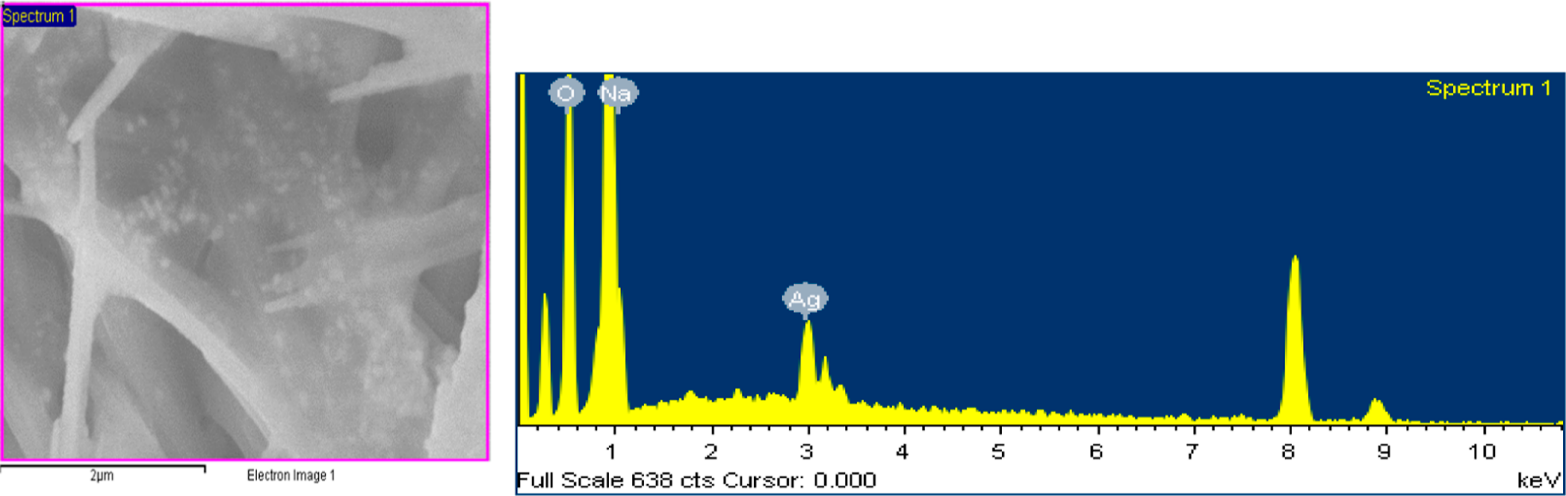
SEM-EDS analysis of CuOHNW after AgNPr were deposited.

Figure 4 shows SEM images of CuOHNW before (Figure 4A) and after deposition of AgNPr by the drop casting method, where it is possible to see a higher accumulation of Ag nanostructures on the surface of NWs when NaBH4 is 1.96% v/v (Figure 4B,C) and a low distribution when NaBH_4_ was 3.85% v/v (Figure 4D,E). In this step, the same experimental conditions were used to deposit AgNPr on CuOHNW for all samples. With Raman spectroscopy, there is not a signal about chemical bonding between AgNPr and Cu foil; however, we found a signal near to 233 cm^–1^ (as can be seen in Figure S1) associated with the Ag–O bond,^26^ suggesting that the metal nanoparticles are bounding to CuOHNWs. A SEM-EDS analysis of a 1.96% v/v sample was implemented, where Ag is confirmed, Figure 5.

**Figure 6:**
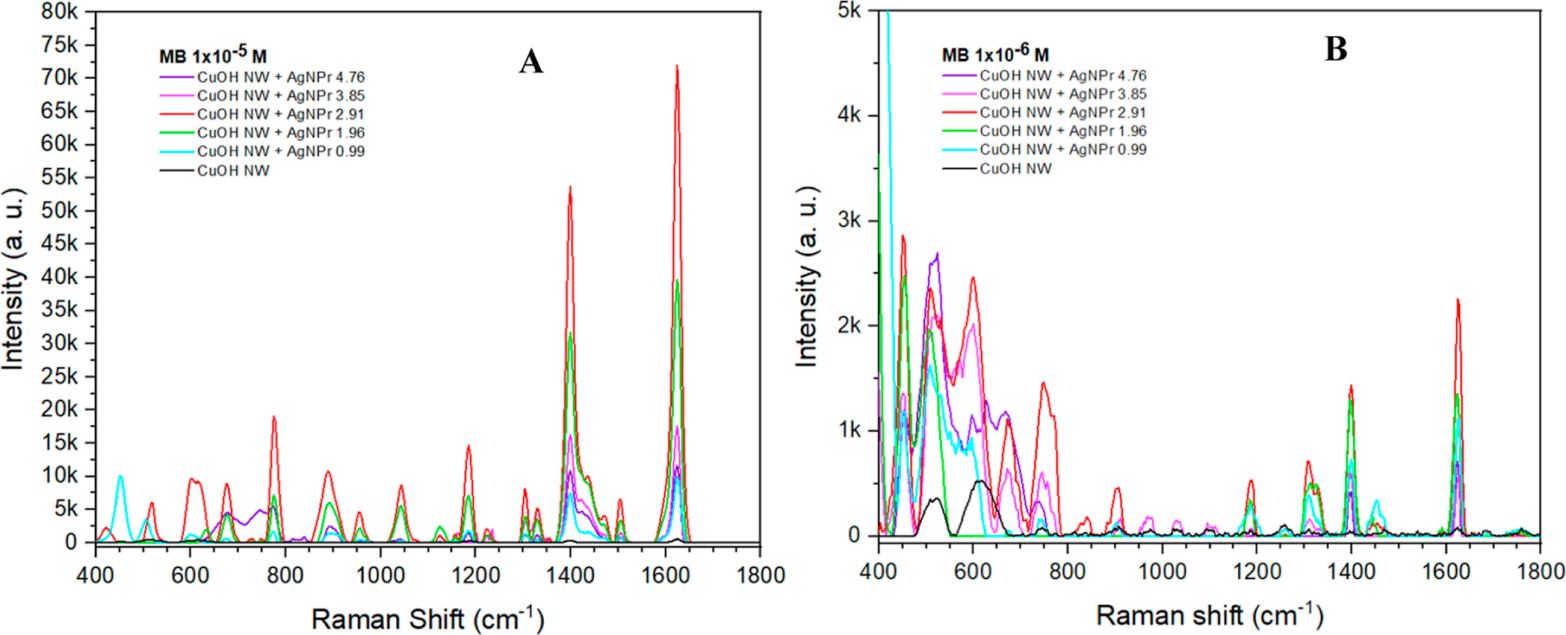
SERS spectra of MB at 1 × 10^–5^ M (A) and 1 × 10^–6^ M (B) concentrations dropped on CuOHNW + AgNPr synthesized with different % v/v of NaBH_4_.

To evaluate the efficiency of AgNPr deposited on CuOHNW as a SERS substrate, two aqueous solutions with 1 × 10^–5^ and 1 × 10^–6^ M concentrations of MB were used as probes. Figure 6A shows the SERS spectra for the 1 × 10^–5^ M concentration, in which several vibrational modes at 1624, 1399, 1303, 1185, 1043, 956, 889, 774, 675, and 600 cm^–1^, associated with MB,^27−29^ are observed. Two prominent peaks around 1624 and 1399 cm^–1^ are assigned to C–C ring stretching and C–N symmetric stretching, respectively. The modes at 1043, 956, 889, and 774 cm^–1^ are associated with bending in the C–H plane, and that at 675 is associated with bending out of the C–H plane. The SERS intensity increases as the NaBH_4_ content increases from 0.99 to 2.91%, where the highest signal is shown. Afterward, the Raman intensity decreases with increasing NaBH_4_ in the nanostructures, showing the lowest signal at 4.76%. Figure 6B shows the SERS spectra of MB, with a concentration of 1 × 10^–6^ M, in which a similar behavior can be observed; the sample obtained with 2.91% shows a higher amplification, but only four modes were observed at 1624, 1399, 1303, and 1185 cm^–1^. For both MB concentrations, the Raman spectra without AgNPr were measured, labeled as CuOHNW (black line), where the lack of the SERS effect is observed. For both MB concentrations, the Raman spectra without AgNPr were measured, labeled as CuOHNW (black line), where the lack of the SERS effect is observed. The increment observed in the SERS spectra on the signal at 400 to 800 cm^–1^ when the MB concentration is 1 × 10^–6^ M is associated with the CuOHNW substrate contribution. As we can see in Figure 6, the spectra of CuOHNW (black line) have two peaks in the same region. Additionally, some changes in the relative intensity of characteristic spectral features due to the interaction of MB molecules with the substrate surface have been observed.^30^

**Figure 7:**
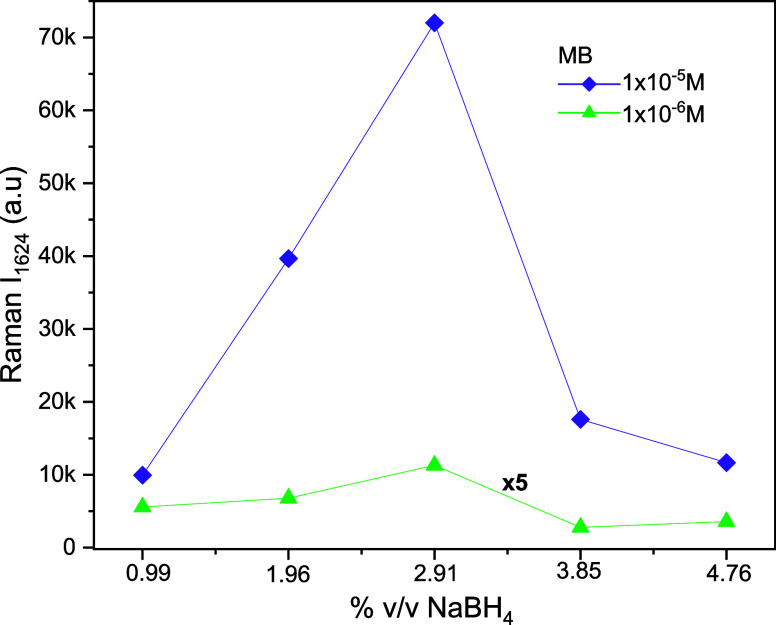
SERS intensity behavior of 1624 mode of MB against the NaBH_4_ concentration used in the AgNPr synthesis.

The intensity of the 1624 cm^–1^ mode for both concentrations can be observed for all samples in Figure 7, where a factor 5× is applied to the intensity of the samples with a concentration of 1 × 10^–6^ M to make it comparable with the 1 × 10^–5^ M intensities.

## Conclusions

In summary, by varying the % v/v of NaBH_4_ during the chemical reduction of AgNO_3_ with sodium citrate, H_2_O_2_ and PVP, we obtained polydisperse triangular AgNPrs with diameters of approximately 60–80 nm in a one-step synthesis. We evaluated these NPrs for 42 days using UV–vis spectrophotometry and ZP, and these colloids maintained their stability. Colloids with lower NaBH_4_ content exhibited shorter reaction times, whereas increasing the volume of NaBH_4_ resulted in longer reaction times. Therefore, it is not advisible to use very high volumes of NaBH_4_, as it will lead to a saturation point and prolong the reaction time. On the other hand, it was possible to relate the coloration of the colloids with the absorption spectrum, morphology, and SERS intensity, where the colloids with lower % v/v of NaBH_4_ showed lighter colorations and were the ones that presented signals with a tendency to increase in SERS; the colloid with the darkest coloration is the one that presented a higher SERS signal, and the last colloids with higher % NaBH_4_ had lower intensity, relating both with a fainter tone to the one with higher intensity. In addition, the effectiveness of the AgNPr on CuOHNW was validated by applying MB detection at a concentration of 1 × 10^–5^ and 1 × 10^–6^ M, where the sample with 2.91% in volume of NaBH_4_ was the one that presented better stability according to the ZP analysis and better SERS amplification.

## Supporting Information Available

Raman spectra of CuOHNWs after AgNPr (PDF)
