# Supplementary material for: Influence of Sodium Borohydride Content on Triangular Silver Nanoprisms Dropped on Copper Hydroxide Nanowire-Based SERS Substrates
Source: ACS Omega. 2024 Nov 15;9(47):46997–7004. doi: 10.1021/acsomega.4c06818 (PMC11603320; doi:10.1021/acsomega.4c06818)
Supplement: Supplementary file 1 — ao4c06818_si_001.pdf [file ao4c06818_si_001.pdf]

Influence of the sodium borohydride content on triangular silver nanoprisms dropped on copper hydroxide nanowires-based SERS substrates

Daniela González-Zárate[1], José Luis Zamora-Navarro[1], María Beatriz de la Mora[2], Guillermo Santana-Rodríguez[3] , Mario Díaz-Solís[4] and Luis Zamora-Peredo[1*]

[1)] Centro de Investigación en Micro y Nanotecnología, Universidad Veracruzana, 94294, Boca del Río, Veracruz, México.
[2)] Instituto de Ciencias Aplicadas y Tecnología, Universidad Nacional Autónoma de México, Ciudad Universitaria, Delegación Coyoacán, 04510, CDMX, Mexico
[3)] Instituto de Investigación en Materiales, Universidad Nacional Autónoma de México, Ciudad Universitaria, Delegación Coyoacán, 04510, CDMX, México.
[4)] Facultad de Ciencias Químicas, Universidad Veracruzana, 94294, Boca del Río, Veracruz, México.
*e-mail: luiszamora@uv.mx

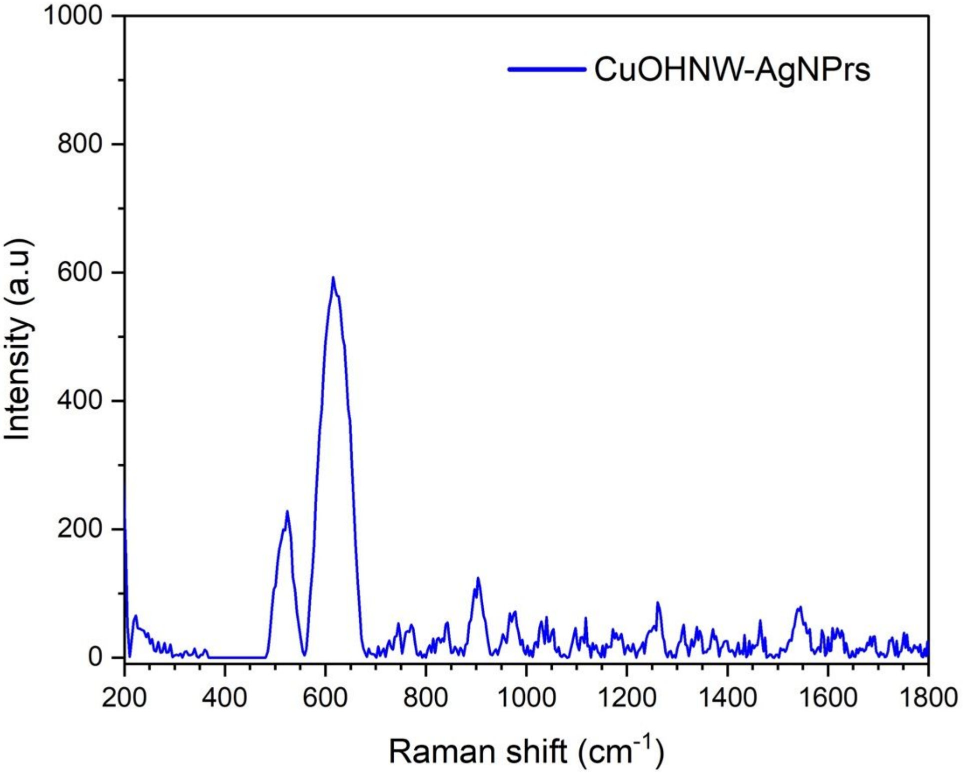

Figure S1. Raman spectra of CuOH nanowires after Ag nanoprisms were deposited.
